# Retrospective Analysis on the Use of Amphotericin B Lipid Complex in Neutropenic Cancer Patients with Suspected Fungal Infections in Lebanon, a Single Center Experience and Review of International Guidelines

**DOI:** 10.3389/fmed.2015.00092

**Published:** 2016-01-05

**Authors:** Rima Moghnieh, Nabila El-Rajab, Dania Issam Abdallah, Ismail Fawaz, Anas Mugharbil, Tamima Jisr, Ahmad Ibrahim

**Affiliations:** ^1^Division of Infectious Diseases, Department of Internal Medicine, Ain WaZein Hospital, Shouf, Lebanon; ^2^Infection Control Program, Ain WaZein Hospital, Shouf, Lebanon; ^3^Department of Internal Medicine, Makassed General Hospital, Beirut, Lebanon; ^4^Faculty of Medical Sciences, Lebanese University, Beirut, Lebanon; ^5^Pharmacy Department, Makassed General Hospital, Beirut, Lebanon; ^6^Division of Hematology-Oncology, Department of Internal Medicine, Makassed General Hospital, Beirut, Lebanon; ^7^Department of Laboratory Medicine, Makassed General Hospital, Beirut, Lebanon

**Keywords:** amphotericin B lipid complex, invasive fungal disease, hematopoietic stem cell transplantation, nephrotoxicity, infusion-related reactions, guidelines

## Abstract

**Introduction:**

Immunocompromised patients carry a high risk for invasive fungal disease (IFD), which is associated with high mortality.

**Materials and methods:**

This is a retrospective chart review of a 4-year experience of amphotericin B lipid complex (ABLC) utilization for the management of suspected IFD at the Hematology/Oncology and Bone Marrow Transplantation unit at Makassed General Hospital, Beirut, Lebanon between January 2011 and December 2014. We focused on treatment strategy, response rate, and adverse drug events associated with ABLC therapy. We also reviewed ABLC indications in international guidelines beyond its Food and Drug Administration approval.

**Results:**

A total of 89 patients received ABLC therapy for suspected fungal infection. Forty-eight percent were treated for a possible fungal infection, 19% for a problable fungal infection, 12% based on hospital guidelines, and 20% based on treating physician’s recommendations. The overall response rate was 71%. Nephrotoxicity occurred in 24% of patients and serum creatinine improved in 10% of these patients. Moderate hypokalemia was observed in 61% of the patients and severe hypokalemia in 10% but was corrected in both cases. Hepatotoxicity was observed in 12% of the patients throughout ABLC therapy. Infusion-related reactions were observed in 36% of the patients. There was a decrease in the incidence of these reactions upon using combination of premedication drugs.

**Conclusion:**

In this study, ABLC proved to be an effective and safe option in the management of suspected IFD in immunocompromised patients failing previous therapies.

## Introduction

Immunocompromised patients, including those on cytotoxic chemotherapy and immunosuppressive regimens, which result in severe and prolonged neutropenia in addition to hematopoietic stem cell transplantation (HSCT) recipients, carry a high risk for invasive fungal disease (IFD), which is associated with a high mortality rate (1, 2). Early diagnosis and aggressive therapeutic approaches to IFD represent important strategies to reduce complications and mortality of these infections (1, 2).

Amphotericin B deoxycholate (conventional amphotericin B) is a polyene with a broad-spectrum activity. It is active against most yeasts, filamentous, and dimorphic fungi. For decades, it has been considered the gold standard therapy against most systemic fungal infections (3). However, the extensive clinical experience obtained with this drug has shown that it is associated with a high risk of treatment limiting toxicity, including infusion-related reactions (IRRs), such as fever and chills, and nephrotoxicity (3). Concerns about its toxicity have led to the development of lipid-based formulations with reduced toxicity, increased therapeutic utility, and significant advance in drug delivery (4).

Three lipid formulations of amphotericin B have been developed and approved for the treatment of systemic fungal infections, two of which amphotericin B lipid complex (ABLC; Abelcet^®^) and liposomal amphotericin B (L-AMB; AmBisome^®^) are currently in widespread use, in addition to amphotericin B colloidal dispersion (ABCD). The lipid composition of all three of these preparations differs considerably and contributes to substantially different pharmacokinetic parameters. ABCD has a similar or higher frequency of IRRs compared with conventional amphotericin B deoxycholate. This high frequency of IRRs resulted in discontinuation of at least one clinical trial and subsequently ABCD has been removed from the commercial market (5).

Amphotericin B lipid complex contains a high ratio of amphotericin B to lipid, in ribbon-like aggregates distinct from liposomes (6). Many studies compared ABLC and L-AMB. There was a considerable heterogeneity among the studies, and the major conclusion was that they were comparable except for higher IRRs with ABLC compared to L-AMB (7). In this respect, Craddock et al. (8) showed a marked decrease in IRRs reaction with ABLC while using premedications along with slow infusion rate, and even recommended a therapeutic algorithm that helps decreasing the rate of IRRs with minimal steroid use (8).

The aim of our study is to retrospectively review a 4-year experience of ABLC (Abelcet; Cephalon Ltd., Herts, UK) utilization for the management of suspected fungal infections in a single center in Lebanon. We looked for the strategy of initiating ABLC therapy with respect to clinical characteristics and risk factors for IFD, clinical response to ABLC therapy, all-cause mortality, along with adverse events associated with the use of ABLC.

Amphotericin B lipid complex was used in this study based on guidelines recommendations and on several comparative studies evaluating safety, efficacy, and cost-effectiveness of ABLC compared to other formulations of amphotericin B (1, 7, 9). It has been proven that 5 mg/kg ABLC delivers the highest tissue concentration of amphotericin B in the liver, spleen, lung, and brain compared to other formulations except in the renal tissue (10–12).

We also reviewed ABLC indications in different international guidelines beyond its original Food and Drug Administration (FDA) approval (refer to Table 1). Its use in different studies has been evaluated previously, based on The Collaborative Exchange of Antifungal Research (CLEAR) database, where most of the literature is based on retrospective analysis of patients who received ABLC with a microbiological proof of IFD (1).

**Table 1:** **Summary of recommendations for the use of amphotericin B lipid complex (ABLC) or other lipid formulations in the management of invasive fungal disease according to regional and international guidelines**.

Guidelines	Indication	Strength of recommendation-quality of evidence	Reference
Clinical Practice Guidelines for the Management of Candidiasis: 2009 Update by the IDSA	Candidemia in non-neutropenic patients	A-I^a^	(13)
	Candidemia in neutropenic patients	A-II^a^ (*C. albicans*) B-III^a^ (*C. glabrata*, *C. parapsilosis*, *C. krusei*)	
	Empirical treatment for suspected invasive candidiasis in non-neutropenic patients	B-III^a^	
	Empirical treatment for suspected invasive candidiasis in neutropenic patients	A-I^a^	
	Treatment for neonatal candidiasis	B-II^a^	
ESCMID guideline for the diagnosis and management of *Candida* diseases 2012: adults with hematological malignancies and after HSCT	Empiric therapy to treat possible *Candida* disease	B-I^b^	(14)
	Targeted treatment of invasive candidiasis/candidaemia	C-II^b^	
Clinical practice guidelines for the management of invasive *Candida* infections in adults in the Middle East region: expert panel recommendations	Proven *Candida* infection non-neutropenic patients	A^a^ (alternative)	(15)
	Proven *Candida* infection neutropenic patients	B^a^ (alternative)	
	Suspected *Candida* infection non-neutropenic patients	B^a^ (alternative)	
	Suspected *Candida* infection neutropenic patients	A^a^ (primary)	
Treatment of aspergillosis: Clinical Practice Guidelines of the IDSA	Invasive pulmonary aspergillosis	A-I^a^ (primary)	(16)
		A-II^a^ (alternative)	
	Invasive sinus aspergillosis (if the etiological organism is not known or histopathologic examination is still pending in anticipation of possible sinus zygomycosis)	A-III^a^	
	Tracheobronchial aspergillosis	B-III^a^ (alternative)	
	Aspergillosis of the CNS	B-III^a^ (alternative)	
	*Aspergillus* osteomyelitis and septic arthritis	B-II^a^ (alternative)	
	*Aspergillus* infections of the eye (endophthalmitis and keratitis)	B-III^a^ (primary)	
	Cutaneous aspergillosis	A-I^a^ (alternative)	
	*Aspergillus* peritonitis	B-III^a^ (primary)	
	Renal aspergillosis	C-III^a^	
	Empirical antifungal therapy of neutropenic patients with prolonged fever despite antibacterial therapy and presumptive therapy for invasive aspergillosis	A-I^a^	
	Salvage therapy of invasive aspergillosis	B-III^b^	
Clinical practice guidelines for the treatment of invasive *Aspergillus* infections in adults in the Middle East region: expert panel recommendations	Invasive pulmonary aspergillosis	B^a^ (alternative)	(17)
	Tracheobronchial aspergillosis	C^a^ (alternative)	
	CNPA (subacute invasive pulmonary aspergillosis)	C^a^ (alternative)	
	Aspergillosis of the CNS	C^b^ (alternative)	
	*Aspergillus* infections of the heart (endocarditis, pericarditis, and myocarditis)	C^a^ (primary)	
	*Aspergillus* osteomyelitis and septic arthritis	B^a^ (alternative)	
	*Aspergillus* infections of the eye (endophthalmitis and keratitis)	B^a^ (alternative)	
	Cutaneous aspergillosis	B^a^ (alternative)	
	*Aspergillus* peritonitis	C^a^ (alternative)	
ESCMID and ECMM joint guidelines on diagnosis and management of hyalohyphomycosis: *Fusarium* spp., *Scedosporium* spp., and others	Treatment of *Fusarium* infection	C-III^b^ (limited case reports)	(18)
ESCMID and ECMM joint clinical guidelines for the diagnosis and management of mucormycosis 2013	First-line treatment of mucormycosis in adult patients except CNS Salvage treatment of mucormycosis in adult patients: refractory to prior antifungal therapy, intolerant to prior antifungal, intolerant due to pre-existing renal disease	B-II^b^ B-II^b^	(19)
ESCMID and ECMM joint clinical guidelines for the diagnosis and management of rare invasive yeast infections	Cryptococcus other than *C. neoformans* and *C. gattii*	B-III^a^	(20)
	CNS and severe infection (induction)		
	Non-CNS, not severe infection		
	*Geotrichum candidum*	B-III^a^	
	*Kodamaea ohmeri*	B-III^a^	
	*Malassezia* (severe)	B-III^a^	
	*Pseudozyma* spp.	A-II^a^	
	*Rhodotorula*	A-II^a^	
	*Saccharomyces*	B-III^a^	
	*Saprochaete capitata*	B-III^a^	
	*Sporobolomyces*	C-III^a^	
	*Trichosporon*	D-III^a^	
European guidelines for antifungal management in leukemia and hematopoietic stem cell transplant recipients: summary of the ECIL 5 – 2013 Update	Invasive candidiasis before species identification	B-II^b^	Reference in footnotes^c^
	Invasive Candidiasis (*C. albicans*, *C. glabrata*, *C. parapsilosis*, *C. krusei*)	B-II^b^	
	Invasive aspergillosis (first line)	B-II^b^	
	Invasive aspergillosis (Salvage)	B-II^b^	
	Mucormycosis first line (except CNS and renal failure)	B-II^b^	
	Mucormycosis (Salvage)	B-III^a^	
Clinical Practice Guideline for the Use of Antimicrobial Agents in Neutropenic Patients with Cancer: 2010 Update by the IDSA	Invasive aspergillosis	A-III (mold-active agent)	(21)
	Anticipated prolonged neutropenic periods of at least 2 weeks	C-III (mold-active agent)	
	Prolonged period of neutropenia immediately prior to HSCT (C-III)	C-III (mold-active agent)	

*KEY: CNS, central nervous system; ECIL, European Conference on Infections in Leukemia; ECMM, European Confederation of Medical Mycology; ESCMID, European Society for Clinical Microbiology and Infectious Diseases; HSCT, hematopoietic stem cell transplantation; IDSA, Infectious Diseases Society of America*.

*N.B. Please refer to each corresponding guidelines for the grading system*.

*^a^Lipid formulation of amphotericin B*.

*^b^Amphotericin B lipid complex*.

^c^http://www.kobe.fr/ecil/telechargements2013/ECIL5%20Antifungal%20Therapy.pdf

## Materials and Methods

This is a retrospective chart review conducted at Makassed General Hospital, a 200-bed university hospital located in Beirut, Lebanon with a 40-bed Hematology/Oncology and Bone Marrow Transplantation unit, between January 2011 and December 2014. It included 89 adult neutropenic cancer patients and HSCT recipients who received at least two doses of ABLC (5 mg/kg/day). The hospital’s Institutional Review Board approved this study, and an informed consent was waived without patient consent due to its observational nature. We recorded demographic data and baseline clinical characteristics; strategy of treatment; use of antifungals prior to ABLC therapy; tolerability and adverse drug events (ADEs) associated with ABLC, including IRRs, nephrotoxicity, hypokalemia, and hepatotoxicity; and premedication combinations used in the prevention of IRRs. Then, we evaluated clinical response to therapy and mortality among these patients.

### Antifungal Prophylaxis

During the study period, antifungal prophylaxis was prescribed according to hospital protocol based on two guidelines: the Third European Conference on Infections in Leukemia (ECIL-3) 2009 guidelines for antifungal management in leukemia and HSCT recipients (22) and the 2011 National Comprehensive Cancer Network (NCCN) clinical practice guidelines in prevention and treatment of cancer-related infections (23). Risk stratification to fungal infections is based on several factors, including underlying malignancy, whether disease is in remission, duration of neutropenia, prior exposure to chemotherapy, and intensity of immunosuppressive therapy. High-risk patients including those with leukemia undergoing induction/salvage chemotherapy and allogeneic HSCT recipients received prophylactic voriconazole (IV 6 mg/kg every 12 h × 2 doses, then 4 mg/kg every 12 h; oral 200 mg PO BID). Recently, in 2014, posaconzaole (200 mg PO TID) was prescribed for this category of patients as per updated hospital protocol. Intermediate-risk patients including autologous HSCT recipients with mucositis only received prophylactic fluconazole or micafungin. Patients with chronic lymphocytic leukemia were prescribed fluconazole (400 mg IV/PO daily) in cases of prolonged neutropenia for >7 days or old age. Patients with lymphoma or multiple myeloma were not put on an antifungal prophylaxis regimen except fluconazole (400 mg IV/PO daily) when there was evidence of oral and/or esophageal candida infections. Low-risk patients including those who receive standard chemotherapy regimens for most solid tumors and patients with anticipated neutropenia for <7 days were not given any prophylactic antifungals.

### Diagnosis and Management

#### Diagnostic Workup

In patients with persistent fever at 72–96 h in the absence of focus of infection with relevant signs and symptoms, a chest X-ray is done and two sets of blood culture are withdrawn, one from central vascular access, and another from peripheral access. If no central vascular access is present, one set of blood culture is taken from peripheral access. A chest computed tomography scan is done if there is a new onset of cough or any new findings on lung exam. Sinuses CT scan is also done in case of any suggestive sign or symptom. In our institution, routine CT scans of chest and sinuses are not always done in persistently febrile neutropenic patients, in order to avoid moving these patients outside from the oncology ward, where the risk of exposure to dust might be higher. Bronchoalveolar lavage (BAL) is done in case of positive finding on chest CT scan and BAL fluid is sent for bacterial and fungal culture; however, the yield of fungal cultures in our institution is low due to technical difficulties. Serum galactomannan levels are recommended in the hospital protocol to be taken twice per week in persistently febrile neutropenic patients; however, it has not been consistently available in the hospital and the country during the study period. So, it was done as per hospital protocol when available.

#### Management

In our study, ABLC utilization was based on three things: The European Organization for Research and Treatment of Cancer/Invasive Fungal Infections Cooperative Group and the National Institute of Allergy and Infectious Diseases Mycoses Study Group (EORTC/MSG) classification of IFD (24), hospital-based protocol for those who do not fit the EORTC/MSG criteria, and treating physician recommendations based on the patient’s condition.

With respect to EORTC/MSG categories, we had two levels of probability to diagnose IFD, which were “probable” and “possible” IFD. Probable IFD requires the presence of a host factor, a clinical criterion, and a mycological criterion. These cases are treated pre-emptively. Others that meet the criteria for a host factor and a clinical criterion but for which mycological criteria are absent are considered possible IFD and are thus treated empirically (24). Host factors include the following: recent history of prolonged neutropenia, receipt of an allogeneic HSCT, prolonged use of corticosteroids, and treatment with T cell immunosuppressants (24). Clinical criteria must be consistent with the mycological findings, if any, and must be related to current episode and confirmed by radiological investigations (24). Mycological criteria include direct testing through identification of fungal elements suggesting molds or recovery by culturing samples from sputum, bronchoalveolar lavage fluid, bronchial, or sinus aspirate; in addition to indirect tests such as serum galactomannan in cases of aspergillosis (24).

According to the hospital protocol, febrile yet clinically stable patients, at 72–96 h after the onset of fever having no focus of infection and negative serum galactomannan, were treated with caspofungin. ABLC was initiated in case of persistent neutropenic fever for ≥4 days in a patient receiving appropriate antibiotic therapy in cases of (1) infiltrates or nodules on chest CT scans/X-Ray or suspected sinusitis based on sinus CT scan ± positive serum galactomannan in patients who were previously on mold-active azole prophylaxis (voriconazole), (2) clinical instability in high-risk patients with previous mold-active prophylaxis (voriconazole), and (3) autologous HSCT recipients with mucositis who are already on micafungin prophylaxis with negative serum galactomannan.

### Response

Clinical success was defined as resolution of all pretreatment signs and symptoms of suspected fungal infection by the end of therapy confirmed by radiological and serological investigations (4, 25). Clinical failure was defined as the progression of disease or the lack of significant improvement or worsening of the same parameters including death the of the patient or drug withdrawal with evidence of ongoing infection after 7 days of antifungal therapy or onset of toxicity that would require discontinuation of the drug (4, 25).

### Adverse Events

Nephrotoxicity was defined as a twofold increase in serum creatinine anytime above baseline during ABLC therapy (25). Improvement in renal function was defined as a decrease in serum creatinine level from a baseline value of ≥1.5 mg/dL to within the normal range or else a >20% decrease from the baseline value (4). Hepatotoxicity was defined as a threefold increase in hepatic transaminases anytime above baseline during ABLC therapy (25). Hypokalemia was defined as decrease in K^+^ level to <3.5 mEq/L. Moderate hypokalemia was having K^+^ level (>2.5–3.5 mEq/L) and severe hypokalemia <2.5 mEq/L (25). Reversible/correctable hypokalemia was defined as K^+^ level increasing to >3.5 mEq/L during ABLC treatment (25) through intravenous and/or oral potassium salt supplementation as per hospital protocol.

### Statistical Analysis

Data were analyzed using SPSS version 19 (SPSS, Chicago, IL, USA). Descriptive statistics and frequencies were performed to obtain percentages. Chi square test was used to assess any significant difference the groups. *P*-value <0.05 was considered significant.

## Results

### Patients’ Characteristics, Treatment Strategy, and Outcome

This study included 89 adult neutropenic cancer patients who received an ABLC dose of 5 mg/kg/day. The mean patient age was 43 years and 56% of the patients were males. In our series, no cases of proven fungal infections were diagnosed. Forty-three patients (48%) fulfilled the EORTC-MSG criteria of possible fungal infection and were treated empirically. Seventeen patients (19%) were treated preemtively for probable fungal infection. Twenty-nine patients (33%) did not fall under any category of the EORTC-MSG classification; yet, they were prescribed ABLC according to hospital guidelines or by their treating hematologist/oncologist because of their either critical condition or persistence of fever in spite of empirical antibiotic therapy in addition to echinocandin or voriconazole prophylaxis. Fifty-seven patients (64%) showed evidence of documented infections like colitis and 19 patients (21%) showed evidence of cytomegalovirus infection identified through viral polymerase chain reaction. In these patients, ABLC was not discontinued because of the assumption of the presence of more than one infection in such a severely ill category of patients. Thus, they were not excluded from the outcome analysis. No routine CMV-PCR is done to all patients except to those who develop colitis or have persistent fever in spite of appropriate antimicrobial treatment. The overall success rate was 71% and total mortality reached 29%. The calculated mortality is crude all-cause mortality not restricted to fungal infection as etiology (refer to Table 2).

**Table 2:** **Clinical characteristics, diagnosis, treatment strategy, and outcome of patients receiving amphotericin B lipid complex therapy**.

Patients’ characteristics	Number of patients (*n* = 89%)
**Age (years)**	
<20	3 (3.4%)
(20–40)	33 (37.1%)
(40–60)	43 (48.3%)
>60	9 (10.1%)
**Gender**	
Male	50 (56.2%)
Female	39 (43.8%)
**Tumor type**	
Leukemia and myelodysplastic disorders on chemotherapy	37 (41.6%)
Lymphoma and other malignancies on chemotherapy	16 (18%)
Autologous HSCT	20 (22.5%)
Allogeneic HSCT	16 (18%)
Graft versus host disease	6 (6.7%)
Central venous catheterization	63 (70.8%)
Mechanical ventilation	19 (21%)
Colitis	57 (64%)
Cytomegalovirus infection	19 (21.3%)
**Diagnosis and management**	
Based on EORTC-MSG classification of IFD^a^	
Possible fungal infection treated empirically	43 (48.3%)
Probable fungal infection treated pre-emptively	17 (19.1%)
Outside the EORTC-MSG classification of IFD	
ABLC therapy based on hospital protocol^b^	11 (12.4%)
ABLC therapy based on treating physician’s recommendations^c^	18 (20.2%)
**Antifungal use prior to ABLC**^d^	
None	33 (37.1%)
Fluconazole	31 (34.8%)
Voriconazole	10 (11.2%)
Posaconazole	3 (3.4%)
Echinocandin	22 (24.7%)
**Response**	
Success	63 (70.8%)
Failure	26 (29.2%)
**Mortality**	
Total mortality	26 (29.2%)
30-day post-treatment mortality	11 (12.4%)

*KEY: ABLC, amphotericin B lipid complex; EORTC-MSG, European Organization for Research and Treatment of Cancer/Invasive Fungal Infections Cooperative Group and the National Institute of Allergy and Infectious Diseases Mycoses Study Group; HSCT, hematopoietic stem cell transplantation; IFD, invasive fungal disease*.

*N.B. ^a^EORTC-MSG classification of IFD is based on the three elements: host factors, clinical manifestations, and mycological evidence*.

*^b^ABLC is initiated in case of persistent neutropenic fever for ≥4 days despite of antibiotic therapy in case of (1) infiltrates or nodules on chest computed tomography (CT) scans/X-Ray or suspected sinusitis based on sinus CT scan ± positive serum galactomannan in patients who were previously on mold-active azole prophylaxis, (2) clinical instability in high-risk patients with previous mold-active prophylaxis, and (3) autologous HSCT recipients with mucositis who are already on micafungin prophylaxis with negative serum galactomannan*.

*^c^Treating physician recommendations to avoid potential infectious complications in such a sick category of patients*.

*^d^More than one agent may be used at different times*.

### Adverse Events

#### Nephrotoxicity

**Figure 1:**
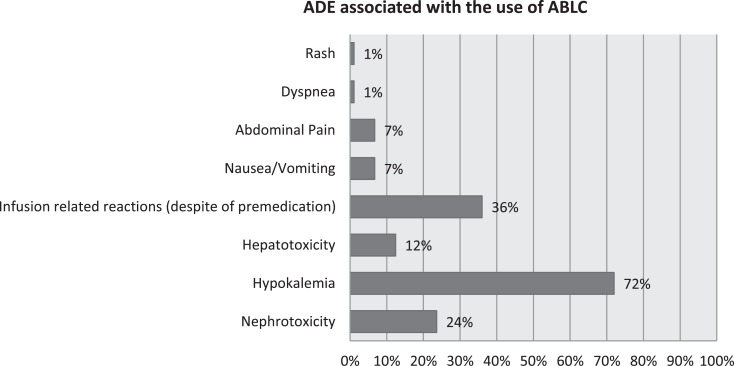
**Adverse drug events (ADEs) associated with the use of amphotericin B lipid complex (ABLC)**.

Nephrotoxicity occurred in 21/89 patients (23.6%). Out of these patients 18/89 (20.2%), had initially a baseline serum creatinine below 1 mg/dL and 3/89 (3.4%) had a baseline serum creatinine above 1 mg/dL. Serum creatinine improved in 3/89 (3.4%) of the whole population and remained persistently elevated in 18/89 (20.2%) of the patients. All of our patients had conditions predisposing to renal impairment, including intake of nephrotoxic anti-infectives and cytotoxic chemotherapeutic agents. Nephrotoxicity was managed by increasing hydration, stopping other concomitantly administered nephrotoxic medication, mostly aminoglycosides whenever possible. In our series, ABLC was not stopped in any of the cases due to persistently elevated serum creatinine according to benefit-risk ratio (refer to Table 3 and Figure 1).

**Table 3:** **Nephrotoxicity due to amphotericin B lipid complex (ABLC)**.

	Baseline serum creatinine <1 mg/dL		Baseline serum creatinine >1 mg/dL	
	% of each category^a^	% of total (*n* = 89)^b^	% of each category^a^	% of total (*n* = 89)^b^
Total	80/80 (100%)	80/89 (88.6%)	10/10 (100%)	10/89 (11.2%)
Doubling serum creatinine at anytime of ABLC therapy	18/80 (22.5%)	18/89 (20.2%)	3/10 (30%)	3/89 (3.4%)
Serum creatinine back to baseline at anytime of ABLC therapy	3/18 (16.7%)	3/89 (3.4%)	0	0
Persistent elevation of serum creatinine	15/18 (83.3%)	15/89 (16.9%)	3/3 (100%)	3/89 (3.4%)
Improving serum creatinine at the end of therapy^c^	1/18 (5.6%)	1/89 (1.1%)	0	0

*N.B. Percentages were calculated in two ways*.

*^a^The denominator was the total number of patients in each subgroup*.

*^b^The denominator was the total number of patients in the study*.

*^c^Improvement in renal function was defined as a decrease in serum creatinine level from a baseline value of ≥1.5 mg/dL to within the normal range or else a >20% decrease from the baseline value*.

#### Hypokalemia

Hypokalemia occurred in 64/89 patients (72%) of which 60.7% (54/89 patients) was moderate hypokalemia and 10.1% (10/89 patients) was severe. Hypokalemia was reversible in 56/89 patients (62.9%) through intravenous and oral potassium salts supplementation as per hospital guidelines. Eight out of 89 patients (9%) failed to get serum potassium levels back to normal and three of them had ABLC discontinued (refer to Table 4 and Figure 1).

**Table 4:** **Hypokalemia due to amphotericin B lipid complex (ABLC)**.

Serum potassium	No hypokalemia (>3.5 mEq/L)		Moderate hypokalemia (>2.5–3.5 mEq/L)		Severe hypokalemia (<2.5 mEq/L)	
	% of each category^a^	% of total (*n* = 89)^b^	% of each category^a^	% of total (*n* = 89)^b^	% of each category^a^	% of total (*n* = 89)^b^
Total	25/25 (100%)	25/89 (28.1%)	54/54 (100%)	54/89 (60.7%)	10/10 (100%)	10/89 (10.1%)
Reversible/correctable	0	0	47/54 (87%)	47/89 (52.8%)	9/10 (90%)	9/89 (10.1%)
Irreversible/not correctable	0	0	7/54 (13%)	7/89 (7.9%)	1/10 (10%)	1/89 (1.1%)
Discontinuation of ABLC due to hypokalemia	0	0	2/54 (3.7%)	2/89 (2.2%)	1/10 (10%)	1/89 (1.1%)

*N.B. Percentages were calculated in two ways*.

*^a^The denominator was the total number of patients in each subgroup*.

*^b^The denominator was the total number of patients in the study*.

#### Hepatotoxicity

Eleven (12.4%) patients out of 89 showed a threefold increase in hepatic transaminases (AST, aspartate aminotransferase and ALT, alanine aminotransferase) levels above baseline throughout ABLC therapy (refer to Figure 1).

#### Infusion-Related Reactions

All patients were premedicated prior to ABLC therapy, yet infusion reactions including fever, chills, and rigors were observed in 32/89 (36%). Several combinations of premedication drugs were used including intravenous rapid acting corticosteroids; steroids and paracetamol; steroids, paracetamol, and antihistamines. Among the patients premedicated with steroids alone, 3/23 (13%) patients developed IRRs. Whereas patients premedicated with steroids and paracetamol, 18/44 (41%) of them developed IRRs. In patients premedicated with steroids, paracetamol and antihistamines, 9/16 (56.3%) developed IRRs (refer to Table 5 and Figure 1).

**Table 5:** **Infusion-related reactions (IRR) associated with amphotericin B lipid complex using different premedication regimens**.

	Premedication protocol				
	Hydrocortisone (H) only	H + Paracetamol (P)	H + Antihistamine (A)	P + A	H + A + P
Number of premedicated patients	23	44	2	2	16
IRR	3	18	1	1	9
%	13	41	50	50	56.3

*N.B. 2/89 patients were not premedicated and no infusion-related reaction occurred*.

*Premedication protocols*.

*IV Hydrocortisone (100–250 mg) alone*.

*IV Hydrocortisone (100–250 mg) + IV Paracetamol (1 g)*.

*IV Hydrocortisone (100–250 mg) + Antihistamine: PO Hydroxyzine HCl (10 mg) or PO Loratidine (10 mg)*.

*IV Paracetamol (1 g) + Antihistamine: PO Hydroxyzine HCl (10 mg) or PO Loratidine (10 mg)*.

*IV Hydrocortisone (100–250 mg) + IV Paracetamol (1 g) + Antihistamine: PO Hydroxyzine HCl (10 mg) or PO Loratidine (10 mg)*.

#### Others

Considering other ADEs in our patient population, 6/89 (6.7%) experienced nausea and vomiting, 6/89 (6.7%) abdominal pain, 1/89 (1.1%) dyspnea, and 1/89 (1.1%) developed skin rash (refer to Figure 1).

## Discussion

This is a retrospective chart review evaluating the use of ABLC in a single center in Lebanon. ABLC was used in this study based on guideline recommendations and on several comparative studies evaluating safety, efficacy, and cost-effectiveness of ABLC compared to other formulations of amphotericin B (1, 7, 9). The Food and Drug Association (FDA) approved ABLC in 1995 for the treatment of invasive fungal infections in patients who are refractory to or intolerant of conventional amphotericin B therapy (26). It appears, with mostly (B-II), (C-II), and sometimes (A-I) or (A-II) levels of recommendation and quality of evidence, in regional and international clinical practice guidelines for the management of invasive aspergillosis (16, 17, 27), invasive candidiasis in neutropenic and non-neutropenic patients (13–15), febrile neutropenia in cancer and HSCT patients (21, 22), and in the guidelines for the diagnosis and management of non-*Aspergillus* molds (18, 19). These indications are beyond the FDA approval. Refer to Table 1 for further information about ABLC indications in regional and international guidelines.

In our study, which included 89 adult patients with neutropenic fever, the overall response rate was 71%. This result was better compared to a subset of patients with presumed fungal infection treated with ABLC in a retrospective study by Mehta et al. (28) where the response rate reached 63%. Our study population is comparable to this subset of patients in this study, which included 64 adult neutropenic cancer patients who were immunocompromised after HSCT or chemotherapy. They received ABLC (5 mg/kg/day), with a total of 68 courses of ABLC therapy (16/68 courses for confirmed cases and 52/68 courses for presumed fungal infections) (28). In another key clinical trial on 556 patients conducted by Walsh et al. (4), the response rate was much less than ours (57%). The main difference between our patient population and Walsh et al. (4) population is that ABLC was used in our study to treat fungal infections in immunocompromised patients in indications outside FDA approval but mentioned in several regional and international guidelines, whereas Walsh et al. (4) used it strictly in patients intolerant or refractory to conventional amphotericin B, as per FDA approval.

The safety and tolerability profile represent a major issue that affects the choice of antifungal therapy. In our study, nephrotoxicity occurred in 24% of the patients; yet, ABLC was not discontinued in any of the cases according to benefit–risk ratio knowing that all of our patients were on multiple nephrotoxic drugs. Nephrotoxicity is the most clinically significant adverse reaction of amphotericin B and the concomitant use of nephrotoxic agents (such as aminoglycosides, cyclosporine) or corticosteroids, cytotoxic chemotherapy is a well-known risk factor for amphotericin B-induced nephrotoxicity (7). In a pharmacovigilance study conducted in Spain involving 93 oncology/hematology patients, no differences in major renal, hematological, and liver function parameters (serum creatinine, hemoglobin, potassium, transaminases, and bilirubin) were reported with ABLC when comparing pretreatment and post-treatment values (29). In a study by Alexander and Wingard on renal safety in ABLC-treated patients, nephrotoxicity was observed in 13% of subjects despite the high risk of renal impairment carried by these patients (30). Two meta-analyses of clinical efficacy and tolerability data, which compared lipid-based formulations of amphotericin B with conventional amphotericin B, concluded that the former are associated with less nephrotoxicity and hypokalemia compared to conventional amphotericin B (31, 32). However, partially due to controversial results and the heterogeinity of the studies, these meta-analyses failed to show any significant difference in renal safety between the different lipid-based formulations of amphotericin B (31, 32). In a literature review of published data on the safety, efficacy, and cost-effectiveness of ABLC, the author concluded that ABLC has a superior tolerability profile compared to conventional amphotericin B, and he also declared that ABLC and L-AmB have a similar risk of nephrotoxicity (9). In a recent review and meta-analysis that compared the drug-induced nephrotoxicity associated with either ABLC or L-Amb, the authors reported an increased probability of nephrotoxicity in patients who were treated with ABLC as compared with L-AmB [odd’s ratio (OR), 1.75; relative risk (RR) 1.55] (33). This was due to the significant lack of homogeneity across these studies, where the results were heavily influenced by an unexplained high rate of nephrotoxicity in one particular study by Wingard et al. (34). When that study was removed from the analysis, the risk of nephrotoxicity was more similar between the two preparations (OR, 1.12; RR, 1.09) (33).

Hypokalemia secondary to urinary potassium wasting is a frequent adverse effect of amphotericin B therapy, where serum potassium levels should be routinely monitored (7). In our study, moderate hypokalemia was observed in around 61% of the cases and severe hypokalemia in 10%. Serum potassium levels were correctable in 63% of patients in both groups by supplying intravenous and oral potassium salts as per hospital guidelines. ABLC was discontinued due to hypokalemia in three patients only. According to a study by Clark et al. (35), electrolyte abnormalities were present in 18/36 (50%) patients on ABLC who experienced a fall in serum potassium levels on therapy to <3 mmol/L. Serum potassium should be routinely monitored with amphotericin B formulations since it has been clearly documented that it induces renal potassium wasting and can produce substantial potassium deficit (35).

Infusion-related reactions, such as fever and chills, which occur with ABLC, are generally mild to moderate and usually last for only 2–3 days after the onset of therapy. IRRs are not dose related and generally diminish with subsequent infusions (36). In our study, the overall rate of IRRs was 36% despite of premedication along with a slow infusion rate that was not standardized during the whole study period. Several combinations of premedication drugs were used including intravenous rapid acting corticosteroids alone; steroids and paracetamol; steroids, paracetamol, and antihistamines all together. Recent studies have highlighted the importance of premedication regimens combined with a reduction in the infusion rate to minimize, or even prevent, the onset of IRRs, which are based on the administration of systemic corticosteroids, paracetamol, with or without chlorphenamine (8). The reported incidence of IRRs with ABLC has ranged between 2 and 23% in several studies (28, 29). It has been postulated that slowing the speed of the ABLC infusion, i.e., to run the dose over 3–4 h has been proven in the literature to decrease the rate of IRRs (8). IRRs are common to all lipid-based formulations of amphotericin B, although L-AMB has been shown to result in a lower incidence than ABLC (37). Yet, they can be easily managed through a combination of premedication and reducing the infusion rate of ABLC. In a study by O’Connor and Borley (38), 100 mg of hydrocortisone was used as premedication 15–30 min prior to ABLC infusion. This resulted in a lower incidence of IRRs than had been reported in published literature for ABLC, 15.3% for the initial infusion and 2.3% for subsequent infusions (38). Craddok et al. (8) suggested a consensus panel algorithm on premedication and infusion rate to reduce the risk of IRRs following ABLC infusion.

There are few reports in the literature of ABLC-induced hepatotoxicity (39). In our study, we observed that 12.4% patients out of 89 showed a threefold increase in hepatic transaminases levels above baseline throughout ABLC therapy. However, it is rare as shown by Hashem et al. (25) where it was observed in 4/52 patients (7.7%) who received ABLC as primary therapy for the treatment of invasive aspergillosis.

This study has a major limitation that it is only descriptive and retrospective. No comparison was made to other antifungals or a control group in addition to the heterogeneity of our patient population including different categories of risk to fungal infections. Although the immunological response in this heterogeneous group of patients differs from one clinical condition to another, it is still comparable to earlier studies that evaluated the efficacy and tolerability of polyene antifungals (4, 28). Fungal attributable mortality was not discussed because of the complexity of the cases even if it maybe partially attributed to fungal infection. Yet, the basic illness, severity of immunosuppression, and thrombocytopenia would have played a major role in the etiology of mortality and can be major confounding factors, add to this that autopsies were not done in this specific population, where family consent can rarely be obtained. In addition, an ideal study would include microbiological confirmation of fungal infection before starting antifungal therapy. The lack of central fungal laboratories in Lebanon, the absence of institutional laboratory techniques for fungus identification, and antifungal susceptibility testing make most antifungal treatment based on clinical assessment.

## Conclusion

Our results showed a higher efficacy of ABLC to what has been previously mentioned in the literature with a comparable toxicity profile for the management of suspected IFD in immunocompromised patients failing previous therapies. Our data were based on empiric or preemptive therapy due difficulty in taking tissue biopsies in such immunocompromised category of patients. Fungus identification as well as antifungal susceptibility testing has become an important tool for physicians in making difficult treatment decisions regarding management of patients with fungal infections, especially in the era of changing epidemiology and drug susceptibility patterns of both candida and mold infections (40). So, we recommend putting efforts in this issue and ultimately in developing an institutional antifungal stewardship program, which will preserve our antifungal armamentarium.

## Conflict of Interest Statement

The authors declare that the research was conducted in the absence of any commercial or financial relationships that could be construed as a potential conflict of interest.

## References

[1] Chu P Sadullah S. The current role of amphotericin B lipid complex in managing systemic fungal infections. Curr Med Res Opin (2009) 25(12):3011–20. 10.1185/03007990903379077 19849324

[2] Chamilos G Luna M Lewis RE Bodey GP Chemaly R Tarrand JJ Invasive fungal infections in patients with hematologic malignancies in a tertiary care cancer center: an autopsy study over a 15-year period (1989-2003). Haematologica (2006) 91:986–9. 16757415

[3] Dupont B. Overview of the lipid formulations of amphotericin B. J Antimicrob Chemother (2002) 49(Suppl S1):31–6. 10.1093/jac/49.suppl_1.31 11801578

[4] Walsh TJ Hiemenz JW Seibel NL Perfect JR Horwith G Lee L Amphotericin B lipid complex for invasive fungal infections: analysis of safety and efficacy in 556 cases. Clin Infect Dis (1998) 26:1383–96. 10.1086/516353 9636868

[5] Timmers GJ Zweegman S Simoons-Smit AM van Loenen AC Touw D Huijgens PC. Amphotericin B colloidal dispersion (Amphocil) vs fluconazole for the prevention of fungal infections in neutropenic patients: data of a prematurely stopped clinical trial. Bone Marrow Transplant (2000) 25(8):879–84. 10.1038/sj.bmt.1702243 10808210

[6] Janoff AS Perkins WR Saletan SL Swenson CE Amphotericin B lipid complex (Ablc^®^): a molecular rationale for the attenuation of amphotericin B related toxicities. J Liposome Res (1993) 3(3):451–71. 10.3109/08982109309150730

[7] Hamill RJ. Amphotericin B formulations: a comparative review of efficacy and toxicity. Drugs (2013) 73(9):919–34. 10.1007/s40265-013-0069-4 23729001

[8] Craddock C Anson J Chu P Dodgson A Duncan N Gomez C Best practice guidelines for the management of adverse events associated with amphotericin B lipid complex. Expert Opin Drug Saf (2010) 9(1):139–47. 10.1517/14740330903418430 19947901

[9] Martino R. Efficacy, safety and cost-effectiveness of amphotericin B lipid complex (ABLC): a review of the literature. Curr Med Res Opin (2004) 20:485–504. 10.1185/030079904125003179 15119986

[10] Wong-Beringer A Jacobs RA Guglielmo BJ. Lipid formulations of amphotericin B: clinical efficacy and toxicities. Clin Infect Dis (1998) 27:603–18. 10.1086/514704 9770163

[11] Vogelsinger H Weiler S Djanani A Kountchev J Bellmann-Weiler R Wiedermann CJ Amphotericin B tissue distribution in autopsy material after treatment with liposomal amphotericin B and amphotericin B colloidal dispersion. J Antimicrob Chemother (2006) 57(6):1153–60. 10.1093/jac/dkl141 16627591

[12] Bekersky I Fielding RM Dressler DE Lee JW Buell DN Walsh TJ. Plasma protein binding of amphotericin B and pharmacokinetics of bound versus unbound amphotericin B after administration of intravenous liposomal amphotericin B (AmBisome) and amphotericin B deoxycholate. Antimicrob Agents Chemother (2002) 46(3):834–40. 10.1128/AAC.46.3.834-840.2002 11850269 PMC127463

[13] Pappas PG Kauffman CA Andes D Benjamin DK Jr Calandra TF Edwards JE Jr Clinical practice guidelines for the management of candidiasis: 2009 update by the Infectious Diseases Society of America. Clin Infect Dis (2009) 48:503–35. 10.1086/596757 19191635 PMC7294538

[14] Ullmann AJ Akova M Herbrech R Viscoli C Arendrup MC Arikan-Akdagli S ESCMID guideline for the diagnosis and management of *Candida* diseases 2012: adults with haematological malignancies and after haematopoietic stem cell transplantation (HCT). Clin Microbiol Infect (2012) 18(Suppl 7):53–67. 10.1111/1469-0691.12041 23137137

[15] Alothman AF Al-Musawi T Al-Abdely HM Salman JA Almaslamani M Yared N. Clinical practice guidelines for the management of invasive *Candida* infections in adults in the Middle East region: expert panel recommendations. J Infect Public Health (2014) 7(1):6–19. 10.1016/j.jiph.2013.08.002 24035607

[16] Walsh TJ Anaissie EJ Denning DW Herbrecht R Kontoyiannis DP Marr KA Treatment of aspergillosis: clinical practice guidelines of the Infectious Diseases Society of America. Clin Infect Dis (2008) 46(3):327–60. 10.1086/525258 18177225

[17] Al-Abdely HM Alothman AF Salman JA Al-Musawi T Almaslamani M Butt AA Clinical practice guidelines for the treatment of invasive *Aspergillus* infections in adults in the middle east region: expert panel recommendations. J Infect Public Health (2014) 7(1):20–31. 10.1016/j.jiph.2013.08.003 24029495

[18] Tortorano AM Richardson M Roilides E van Diepeningen A Caira M Munoz P ESCMID and ECMM joint guidelines on diagnosis and management of hyalohyphomycosis: *Fusarium* spp., *Scedosporium* spp. and others. Clin Microbiol Infect (2014) 20(Suppl 3):27–46. 10.1111/1469-0691.12465 24548001

[19] Cornely OA Arikan-Akdagli S Dannaoui E Groll AH Lagrou K Chakrabarti A ESCMID and ECMM joint clinical guidelines for the diagnosis and management of mucormycosis 2013. Clin Microbiol Infect (2014) 20(Suppl 3):5–26. 10.1111/1469-0691.12371 24479848

[20] Arendrup MC Boekhout T Akova M Meis JF Cornely OA Lortholary O ESCMID and ECMM joint clinical guidelines for the diagnosis and management of rare invasive yeast infections. Clin Microbiol Infect (2014) 20(Suppl 3):76–98. 10.1111/1469-0691.12360 24102785

[21] Freifeld AG Bow EJ Sepkowitz KA Boeckh MJ Ito JI Mullen CA. Clinical practice guideline for the use of antimicrobial agents in neutropenic patients with cancer: 2010 update by the infectious diseases society of America. Clin Infect Dis (2011) 52(4):e56–93. 10.1093/cid/cir073 21258094

[22] Maertens J Marchetti O Herbrecht R Cornely OA Flückiger U Frêre P European guidelines for antifungal management in leukemia and hematopoietic stem cell transplant recipients: summary of the ECIL 3 – 2009 update. Bone Marrow Transplant (2011) 46(5):709–18. 10.1038/bmt.2010.175 20661235

[23] National Comprehensive Cancer Network. NCCN Clinical Practice Guidelines in Oncology Prevention and Treatment of Cancer-Related Infections (v.2.2011). Fort Washington, PA: National Comprehensive Cancer Network (2011).

[24] De Pauw B Walsh TJ Donnelly PJ Stevens DA Edwards JE Calandra T Revised definitions of invasive fungal disease from the European organization for research and treatment of cancer/invasive fungal infections cooperative group and the national institute of allergy and infectious diseases mycoses study group (EORTC/MSG) consensus group. Clin Infect Dis (2008) 46:1813–21. 10.1086/588660 18462102 PMC2671227

[25] Hachem RY Boktour MR Hanna HA Husni RN Torres HA Afif C Amphotericin B lipid complex versus liposomal amphotericin B monotherapy for invasive aspergillosis in patients with hematologic malignancy. Cancer (2008) 112:1282–7. 10.1002/cncr.23311 18224662

[26] Dismukes WE Introduction to antifungal drugs. Clin Infect Dis (2000) 30:653–7. 10.1086/313748 10770726

[27] Herbrecht R Flückiger U Gachot B Ribaud P Thiebaut A Cordonnier C Treatment of invasive *Candida* and invasive *Aspergillus* infections in adult haematological patients. EJC Suppl (2007) 5:49–59. 10.1016/j.ejcsup.2007.06.007

[28] Mehta J Kelsey S Chu P Powles R Hazel D Riley U Amphotericin B lipid complex (ABLC) for the treatment of confirmed or presumed fungal infections in immunocompromised patients with hématologie malignancies. Bone Marrow Transplant (1997) 20(1):39–43. 10.1038/sj.bmt.1700842 9232254

[29] Aguado JM Lumbreras C González-Vidal D Grupo de Farmacovigilancia de Abelcet. Assessment of nephrotoxicity in patients receiving amphotericin B lipid complex: a pharmacosurveillance study in Spain. Clin Microbiol Infect (2004) 10:785–90. 10.1111/j.1198-743X.2004.00963.x 15355408

[30] Alexander BD Wingard JR. Study of renal safety in amphotericin B lipid complex-treated patients. Clin Infect Dis (2005) 40(Suppl 6):S414–21. 10.1086/429335 15809928

[31] Macauly SS Martin JE Zarnke KB Amphotericin B for the treatment of systemic fungal infections: meta-analysis of conventional versus lipid formulations. Interscience Conf on Antimicrobial Agents and Chemotherapy. 42nd ICAAC. San Diego, CA (2002).

[32] Barrett JP Vardulaki KA Conlon C Cooke J Daza-Ramirez P Evans EG A systematic review of the antifungal effectiveness and tolerability of amphotericin B formulations. Clin Ther (2003) 25:1295–320. 10.1016/S0149-2918(03)80125-X 12867214

[33] Safdar A Ma J Saliba F Dupont B Wingard JR Hachem RY Drug-induced nephrotoxicity caused by amphotericin B lipid complex and liposomal amphotericin B: a review and meta-analysis. Medicine (2010) 89(4):236–44. 10.1097/MD.0b013e3181e9441b 20616663

[34] Wingard JR White MH Anaissie E Raffalli J Goodman J Arrieta A A randomized, double-blind comparative trial evaluating the safety of liposomal amphotericin B versus amphotericin B lipid complex in the empirical treatment of febrile neutropenia. Clin Infect Dis (2000) 31(5):1155–63. 10.1086/317451 11073745

[35] Clark AD McKendrick S Tansey PJ Franklin IM Chopra R. A comparative analysis of lipid-complexed and liposomal amphotericin B preparations in haematological oncology. Br J Haematol (1998) 103:198–204. 10.1046/j.1365-2141.1998.00934.x 9792308

[36] Fleming RV Kantarjian HM Husni R Rolston K Lim J Raad I Comparison of amphotericin B lipid complex (ABLC) vs. AmBisome in the treatment of suspected or documented fungal infections in patients with leukemia. Leuk Lymphoma (2001) 40:511–20. 10.3109/10428190109097650 11426524

[37] Tiphine M Letscher-Bru V Herbrect R. Amphotericin B and its new formulations: pharmacologic characteristics, clinical efficacy, and tolerability. Transpl Infect Dis (1999) 1:273–83. 10.1034/j.1399-3062.1999.010406.x 11428998

[38] O’Connor N Borley A Prospective audit of the effectiveness of hydrocortisone premedication on drug delivery reactions following amphotericin B lipid complex. Curr Med Res Opin (2009) 25:749–54. 10.1185/03007990902752753 19196219

[39] Barron RL Kazakoff PW Gabel I Madinger NE Novel hepatotoxicity of amphotericin B lipid complex in combination with cyclosporine A (abstract no LM-61b). In: Program and abstracts of the 37th Interscience Conference on Antimicrobial Agents and Chemotherapy (Toronto). Washington, DC: American Society of Microbiology (1997). 375 p.

[40] Arendrup MC. Update on antifungal resistance in *Aspergillus* and *Candida*. Clin Microbiol Infect (2014) 20(Suppl 6):42–8. 10.1111/1469-0691.12513 24372701

